# Disparities in ACL Injury and Management: The Impact of Sex, Race, and Social Determinants of Health

**DOI:** 10.1007/s12178-026-10021-2

**Published:** 2026-03-17

**Authors:** Paul B. Walker, Babapelumi Adejuyigbe, Isaiah Hoffman, Timothy Dull, Camille Motchoffo Simo, Kristofer J. Jones

**Affiliations:** University of California, Los Angeles, 10833 Le Conte Ave, Los Angeles, CA 90095 USA

**Keywords:** Anterior cruciate ligament, ACL reconstruction, Sex and gender differences, Racial disparities, Socioeconomic status

## Abstract

**Purpose of Review:**

Anterior cruciate ligament (ACL) injury is a common condition that significantly affects physical function and long-term knee health across diverse patient populations. Although outcomes after ACL reconstruction have improved, growing evidence demonstrates persistent disparities related to biological sex, gender, race, ethnicity, and socioeconomic factors. This review synthesizes recent literature examining how these factors influence ACL injury risk and outcomes, with the goal of identifying actionable gaps and future directions to improve equity in ACL management.

**Recent Findings:**

Recent studies confirm that female patients sustain ACL injuries at higher rates and experience inferior patient-reported outcomes and lower return-to-sport rates despite largely equivalent objective surgical outcomes. Recent work highlights neuromuscular and plyometric-based rehabilitation, psychological readiness, and adaptable rehabilitation environments as key modifiable contributors to these disparities. Racial and ethnic minority patients experience delayed surgical care, reduced rehabilitation utilization, and worse functional outcomes, with neighborhood-level socioeconomic deprivation and access-related barriers emerging as key modifiable contributors.

**Summary:**

Disparities in ACL outcomes arise from the interaction of biological risk, rehabilitation-dependent recovery, and structural barriers affecting access to timely surgery and postoperative care. Rehabilitation offers a critical, modifiable opportunity to reduce these gaps by optimizing neuromuscular recovery and ensuring equitable access to supervised therapy. Future progress will require prospective, sex-, race-, or ethnicity-stratified studies, integration of area-level socioeconomic metrics, and care pathways that address delays in evaluation, surgery, and rehabilitation. Earlier implementation of neuromuscular injury-prevention programs in youth and community-based settings may further reduce downstream disparities.

## Introduction

Anterior cruciate ligament (ACL) injury is one of the most common and consequential conditions in sports medicine, affecting an estimated 200,000 patients annually in the United States and generating over $7 billion in direct and indirect healthcare costs [1]. This burden extends well beyond the initial injury, encompassing surgical care, rehabilitation, loss of productivity, and an increased risk of post-traumatic sequelae.

Although advances in surgical technique, rehabilitation, and injury prevention have improved outcomes following ACL reconstruction (ACLR), growing evidence indicates that these benefits are not distributed equitably across patient populations. Biological sex, gender-related factors, race, ethnicity, and socioeconomic metrics influence multiple aspects of ACL management, including injury risk, access to specialty care, rehabilitation utilization, return-to-sport trajectories, and functional and patient-reported outcomes [2–5]. Increasing recognition of these disparities has highlighted critical gaps in current clinical frameworks, many of which were developed using predominantly male or socioeconomically advantaged cohorts and may inadequately account for biological variability or structural barriers to care [6–8]. 

Addressing disparities in ACL management is therefore essential not only to advance equity in musculoskeletal care but also to optimize population-level outcomes and reduce burden on the healthcare system. This review synthesizes evidence on how sex, gender, race, and ethnicity influence ACL injury management, with the aim of identifying key gaps and opportunities to improve care delivery and patient outcomes across diverse populations.

## Sex and Gender Disparities in ACL Injury and Management

In the context of ACL injury and recovery, it is essential to distinguish biological sex from gender as distinct yet interrelated constructs. Biological sex encompasses chromosomal, hormonal, and anatomical attributes that influence ligament morphology, neuromuscular biomechanics, and tissue properties relevant to injury susceptibility and surgical outcomes. In contrast, gender refers to socially constructed roles, norms, behaviors, and identities that shape lived experiences, including patterns of sport participation, healthcare utilization, rehabilitation engagement, and psychological adaptation following injury. Whereas sex-related factors primarily contribute to biological differences in injury risk and graft-related outcomes, gender-related factors more often influence rehabilitation environments, return-to-sport decision-making, and access to care. Conceptual clarity between these constructs is critical to avoid conflating intrinsic biological risk with socially mediated determinants of recovery and long-term functional outcomes.

### Anatomical and Biomechanical Risk Factors by Sex

Female athletes sustain ACL injuries at rates approximately two to eight times higher than their male counterparts, most commonly through noncontact mechanisms [4]. These injuries typically occur during landing, cutting, and deceleration and are associated with reduced hip and knee flexion, increased knee valgus, and greater tibial rotation at initial ground contact [3]. Consistent with this biomechanical profile, a recent prospective study of over 800 elite female athletes identified static knee valgus as an independent risk factor for primary ACL injury and knee hyperextension as an independent predictor of both primary and secondary ACL injury [9]. 

After puberty, the incidence of ACL injury increases markedly among female athletes as post-pubertal females exhibit significantly greater knee valgus angles, external knee abduction moments, and internal rotation during jump-landing tasks compared with pre-pubertal girls [10]. These changes coincide with rapid increases in limb length and body mass during adolescence that often outpace neuromuscular adaptation [11–13]. 

Sex-based anatomical features further increase ACL strain during high-risk activities. Well-established contributors include a smaller ACL cross-sectional area, narrower intercondylar notch morphology (often A-shaped), a greater quadriceps angle, and an increased posterior tibial slope, particularly of the lateral tibial plateau [3, 14, 15]. Biomechanical work has shown that ACL cross-sectional area is a strong predictor of peak ligament strain during simulated pivot-landing tasks, with female knees exhibiting approximately 95% greater peak relative ACL strain than male knees [16]. Emerging evidence also implicates lateral femoral condyle morphology as an additional contributor to ACL injury risk in females. An increased lateral femoral condyle ratio may alter femorotibial articulation and exacerbate pivot-related instability [17–19]. Key anatomical risk factors and supporting evidence are summarized in Table 1.

**Table 1:** Sex-based anatomical factors associated with increased ACL injury risk

Anatomic factor	Sex-based difference in females	Association with ACL injury risk
Quadriceps angle (Q-angle)	Greater Q-angle related to pelvic width and lower extremity alignment	Increased valgus loading and altered frontal-plane mechanics during landing and cutting, leading to higher ACL strain
Intercondylar notch morphology	Narrower notch width and higher prevalence of A-shaped morphology	Narrower notch width independently associated with increased ACL injury risk, particularly in females
ACL size and cross-sectional area	Smaller ACL size and cross-sectional area	Smaller ACL size associated with higher ligament strain and increased injury risk during pivoting activities
Posterior tibial slope	Steeper posterior tibial slope	Increased anterior tibial translation and rotational instability
Lateral femoral condyle morphology	Increased lateral femoral condyle ratio	Altered femorotibial articulation and increased pivot-related instability, especially when combined with other anatomic risk factors

### Hormonal Influences on ACL Injury

Hormones across the menstrual cycle have been implicated in modulating ACL injury. Epidemiologic studies suggest a higher incidence of ACL injury during the preovulatory and ovulatory phases of the menstrual cycle, with several analyses demonstrating peak injury rates around the time of ovulation [20–23]. This association appears attenuated among oral contraceptive users, with large observational studies, including cohorts exceeding 10,000 women, reporting a lower risk of ACL injury and similar protective effects across short- and long-term use [23–25]. 

Population-based analyses further demonstrate lower rates of oral contraceptive use among adolescent females undergoing ACL reconstruction compared with matched controls, supporting a potential protective association in younger athletes [26]. This association may extend across contraceptive delivery methods, including oral, device-based, and systemic administration [27]. In contrast, Wang et al. evaluated risk factors for ACL reconstruction rather than ACL injury and reported higher oral contraceptive use among women who underwent surgery [28]. This observation is likely attributable to age-related differences in contraceptive use and patterns of surgical management, rather than indicating an absence of hormonal influence on ACL injury risk.

Experimental data provide potential mechanistic insight into these clinical observations. In a rat model, oral contraceptive formulations with higher progestin-to-estrogen ratios were associated with significantly increased ACL tensile strength compared with controls, accompanied by increased type I collagen expression and reduced matrix metalloproteinase-1 expression [29]. These findings suggest that hormonal modulation may influence ligament biology and injury susceptibility; however, the clinical relevance of oral contraceptive use remains incompletely defined.

### Sex Differences in Access to Care and Surgical Timing

In addition to biological influences, sex-based differences in ACL outcomes are shaped by variation in access to care and timing of surgical intervention. In German professional soccer, female athletes undergo ACL reconstruction significantly later than male athletes, with a median time from injury to surgery of 19 days for women compared with 6 days for men, despite similar rates of operative management [30]. 

Similar patterns have been reported in other elite athletic populations. Female professional soccer players competing in the top five European leagues demonstrate longer time to return to play following ACL reconstruction than male players, with mean return times of 300 days versus 248 days, respectively [31]. Although these cohorts have access to specialized sports medicine care, the observed delays suggest sex-based differences in treatment pathways. This may contribute to prolonged deconditioning, slower rehabilitation progression, and delayed return to sport.

Notably, evidence for sex-based differences in surgical timing is limited to European professional cohorts, and its generalizability to U.S. healthcare systems and non-elite populations remains unknown. Given that delays beyond five months are associated with increased risks of meniscal and chondral injury [1], this gap represents a clinically meaningful limitation in the current literature.

### Sex Differences in Objective Surgical Outcomes

Objective surgical outcomes after ACLR are largely comparable between men and women. A systematic review of studies from the Multicenter Orthopaedic Outcomes Network (MOON) cohort found no sex-based differences in graft tear rates at 2 or 6 years of follow-up [32]. Rates of contralateral native ACL injury were also similar between males and females, consistent with large registry-based analyses [33]. 

Graft choice appears to be a more important determinant of failure risk than biological sex in younger patients. Among adolescents and young adults, bone-patellar tendon-bone autografts are associated with lower graft rupture rates than hamstring tendon autografts, particularly in female cohorts [34, 35]. When graft type is held constant, graft survival is comparable between males and females. In a study evaluating quadriceps tendon autografts, no significant sex-based differences in graft failure were observed at 24-month follow-up [36]. 

Objective measures of postoperative knee stability are largely comparable between sexes. Although females may demonstrate slightly greater instrumented laxity, reported differences are small (typically < 1 mm) and unlikely to be clinically meaningful [3, 36, 37].

Revision risk at mid-term follow-up is also largely comparable between sexes. In a young athletic cohort with a mean follow-up of 73 months, no significant sex-based differences in revision ACLR or contralateral ACL injury were observed [38]. Meta-analytic data suggest a modest increase in relative revision risk among female patients, although the absolute difference remains small [37]. 

### Sex Differences in Patient-Reported Outcomes and Return to Sport

In contrast to largely equivalent objective surgical outcomes, patient-reported outcomes after ACL reconstruction consistently favor males. Multiple longitudinal studies demonstrate that female patients report worse symptoms, pain, and sports-related function across Knee Injury and Osteoarthritis Outcome Score (KOOS) subscales, with disparities persisting into mid- and long-term follow-up [39, 40]. In a prospective cohort, Bjornsen et al. observed progressive improvement in KOOS Quality of Life scores among males over time, whereas females demonstrated minimal improvement and significantly lower scores at 36, 48, and 60 months postoperatively [39]. Other analyses further identify female sex as an independent predictor of inferior patient-reported outcomes, including lower International Knee Documentation Committee (IKDC) scores, even after adjustment for age, body mass index, concomitant meniscal or chondral pathology, and psychological readiness [41, 42]. These findings indicate that sex-based differences in perceived recovery persist despite comparable objective surgical results.

Return-to-sport outcomes represent one of the most pronounced sex-based disparities following ACL reconstruction. Across multiple follow-up intervals, female patients have approximately 23–25% lower odds of returning to sport than males, with differences evident within the first postoperative year and persisting into mid- and long-term follow-up [5]. Age-stratified analyses suggest an even greater disparity among younger athletes, as females younger than 19 years exhibit a 32% lower likelihood of returning to sport compared with their male counterparts [5]. This gap is particularly evident for return to high-risk pivoting sports, where males consistently demonstrate higher return rates and faster return to both training and competition [32, 43, 44]. 

Beyond return rates alone, female patients also exhibit lower activity levels after return to sport. Longitudinal studies demonstrate persistent associations between female sex and inferior Marx activity scores at 2, 6, and 10 years of follow-up, as well as lower Tegner activity scores [32, 37]. Although one study in a non-elite population reported higher return-to-sport rates among women, this finding contrasts with the broader evidence from athletic cohorts [45]. 

Sex-based differences in return to sport are also influenced by distinct barriers to recovery. Female patients more frequently cite loss of interest, work or family responsibilities, and psychological factors such as fear of reinjury or lack of confidence as reasons for not returning to sport [43, 46]. In contrast, males demonstrate higher psychological readiness, as measured by ACL Return to Sport after Injury scores, suggesting that psychological readiness may contribute meaningfully to observed differences in return-to-sport outcomes [46]. 

Physical recovery trajectories further differ by sex. Female patients demonstrate slower strength recovery and greater persistent quadriceps deficits following ACL reconstruction, with clinically meaningful differences emerging as early as 6 months postoperatively and persisting through at least 24 months. Males consistently achieve higher limb symmetry indices for quadriceps and hamstring strength, as well as greater normalized knee extensor torque [43, 47, 48]. These disparities are particularly pronounced following quadriceps tendon autograft reconstruction, with female patients demonstrating greater quadriceps asymmetry, delayed functional recovery, and lower postoperative activity levels [36, 46, 49]. Further evidence identifies female sex as an independent predictor of persistent quadriceps weakness, a finding that may be underestimated in mixed-sex analyses [50]. 

Rehabilitation appears to be a key, modifiable driver of sex- and gender-based disparities following ACL reconstruction, with growing evidence supporting neuromuscular and plyometric-integrated approaches to improve outcomes in women [5, 51, 52]. In a recent mixed-methods concept-mapping study, seven rehabilitation domains relevant to women after ACL injury were identified, with goal-driven rehabilitation, mental and emotional well-being, and adaptable, supportive rehabilitation environments rated as the most important and feasible strategies for improving outcomes [51]. These domains specifically address neuromuscular and psychological factors linked to delayed recovery and inferior patient-reported outcomes in women. Consistent with this framework, neuromuscular rehabilitation programs incorporating plyometrics produce measurable improvements in landing biomechanics, including reductions in knee valgus and knee abduction moments, improved hop performance and limb symmetry indices, and enhanced dynamic stability [53–55]. In post-ACLR populations, rehabilitation protocols integrating plyometric training have demonstrated superior functional performance at 1- and 2-year follow-up compared with traditional approaches [56]. Accordingly, post-ACL reconstruction rehabilitation should incorporate functional performance testing alongside psychological readiness assessment to support safer return-to-sport decisions.

### Sex-Specific Research Gaps and Priorities

Substantial gaps remain in sex-disaggregated reporting of outcomes and treatment-specific variables in ACLR research. A systematic review of 776 studies found that no investigations simultaneously reported patient-reported outcomes and graft choice stratified by biological sex, and fewer than one-third disaggregated KOOS outcomes by sex alone [8]. This lack of integrated reporting limits understanding of sex-specific recovery trajectories and impedes evidence-based graft selection for female patients.

Current ACL injury prevention, rehabilitation, and return-to-sport protocols are similarly constrained by their reliance on male-dominant datasets. As a result, commonly used protocols inadequately account for sex-specific physiology and gender-related barriers to training adherence and recovery [6, 7]. Studies further highlight the need for sex- and gender-responsive rehabilitation frameworks, identifying key considerations for female patients that include psychological well-being, adaptable training environments, competing work or family responsibilities, and access to appropriate rehabilitation resources [51]. Structural inequities in training infrastructure and equipment access may further contribute to persistent disparities in female athletic recovery [3]. 

Taken together, these limitations suggest that future work should prioritize sex-specific rehabilitation protocols and prospective, sex-stratified studies, alongside earlier implementation of neuromuscular injury-prevention programs during adolescence.

## Racial and Ethnic Disparities in ACL Injury and Management

Compared with sex- and gender-based disparities, fewer studies have examined race, ethnicity, and socioeconomic influences on ACL injury and recovery; however, a growing body of evidence highlights their substantial impact on access to care, rehabilitation, and functional outcomes.

### Genetic and Anatomical Predisposition by Race and Ethnicity

Population-level studies demonstrate higher ACL injury rates among White European American female athletes compared with non-White athletes [57]. Anatomical variation may partially explain this pattern, as African American individuals have wider intercondylar notch dimensions than White individuals of the same sex, with reported mean notch widths of 15.5 mm versus 14.1 mm among women and 18.0 mm versus 16.9 mm among men [58]. This wider notch morphology may confer a biomechanical protective effect. Consistent with these observations, genetic analyses have implicated ancestry in ACL injury susceptibility, with individuals sustaining noncontact ACL injuries demonstrating higher European and lower African ancestry scores than uninjured controls [59]. 

### Structural Barriers to Timely ACL Care

Despite potentially higher baseline injury risk among White athletes, racial and ethnic minority populations experience worse downstream outcomes following ACL injury. Across adult and pediatric populations, Black and Hispanic patients experience longer delays from injury to evaluation and ACL reconstruction compared with White patients. Recent studies demonstrate that these patients have approximately 1.5- to 2-fold higher odds of delayed surgery, even after adjustment for insurance status, income, and neighborhood-level socioeconomic measures [60, 61]. 

These delays are clinically meaningful, as prolonged time from injury to reconstruction is associated with increased rates and severity of meniscal and chondral pathology [62, 63]. Hispanic adult patients demonstrate a higher likelihood of bilateral meniscal injury at surgery [64], while Black and Hispanic pediatric patients are more than twice as likely to sustain irreparable meniscal tears requiring meniscectomy compared with White and Asian patients [65]. Postoperative disparities further reflect structural inequities, with Black and Hispanic pediatric patients attending fewer physical therapy visits and demonstrating greater residual quadriceps and hamstring weakness at 9 months postoperatively [65]. 

Revision and re-rupture risk do not uniformly parallel disparities in access and recovery. Several large database studies have demonstrated higher revision rates among White patients [64, 66], whereas Black patients have been shown to have a lower risk of revision in the under 21-year age group [67]. These differences likely reflect variation in postoperative sport exposure, follow-up care, and re-presentation after reinjury. Accordingly, revision rates alone provide an incomplete measure of outcome equity and should be interpreted alongside functional recovery, rehabilitation utilization, and return-to-activity outcomes.

A notable deficiency of the current ACLR literature is the inadequate reporting and analysis of PROMs stratified by race and ethnicity. A 2022 systematic review identified only 9 studies assessing race or ethnicity in relation to ACLR outcomes, most of which focused on access-related barriers rather than PROMs specifically [66]. In one contemporary cohort, racial composition was described as “heavily skewed” and excluded from statistical analysis, underscoring persistent methodological limitations in this area [67]. Pediatric data demonstrate clear racial disparities; however, standardized PROM differences were not reported [65]. Compounding this limitation, disproportionate loss to follow-up among Black patients introduces attrition bias in PROM analyses, as underrepresentation in postoperative assessments may distort outcome estimates and obscure true disparities [68]. This gap is particularly concerning given broader orthopedic evidence demonstrating racial variation in baseline PROM scores and the infrequent reporting of race and ethnicity in clinical trials (8.5–36% of studies) [69–71]. Prospective studies with systematic race- and ethnicity-stratified PROM reporting are needed to clarify whether observed disparities reflect social determinants of health, measurement bias, or true differences in clinical outcomes.

### Social Deprivation and Neighborhood Disadvantage

Growing evidence suggests that many race and ethnicity-associated disparities in ACL management are mediated by socioeconomic factors rather than race itself. Neighborhood-level deprivation indices, including the Area Deprivation Index (ADI) and Social Deprivation Index (SDI), capture structural barriers related to income, education, employment, housing, and insurance access that disproportionately affect racial and ethnic minority populations [68]. 

Patients residing in the most disadvantaged neighborhoods demonstrate more than twice the risk of delayed ACL reconstruction beyond 12 weeks and 6 months, even after adjustment for race and insurance status, and experience higher rates of concomitant meniscectomy for irreparable meniscal tears [69]. Across multiple cohorts, delays exceeding 3 to 6 months occur in approximately 40 to 50% of patients from high-deprivation neighborhoods compared with 20 to 25% of those from the least deprived areas, accompanied by 1.5- to 2-fold higher rates of irreparable meniscal pathology [70, 71]. Similar patterns are observed in pediatric populations using the Child Opportunity Index, where children from low-opportunity neighborhoods experience longer delays to surgery and higher rates of concomitant meniscal injury independent of race and insurance status [72]. 

The effects of social deprivation extend beyond the timing of surgery. Higher social deprivation is consistently associated with inferior patient-reported outcomes following ACL reconstruction, including worse PROMIS Physical Function and Pain Interference scores, with differences of approximately 4 to 6 points that approach or exceed established minimal clinically important differences [71, 73]. In pediatric populations, severe socioeconomic deprivation is associated with worse psychosocial outcomes at 9 months, including greater pain, fatigue, depression, and anxiety, despite comparable improvements in objective physical function [74]. Disparities in rehabilitation access further compound these outcome differences, as patients from higher-deprivation areas experience delayed initiation of physical therapy, fewer total visits, and shorter overall rehabilitation duration, in part due to limited Medicaid acceptance and prolonged wait times [75, 76]. Although Medicaid-insured patients may achieve comparable early improvements in knee range of motion when able to access care, range of motion alone does not fully capture strength recovery, functional readiness, or return-to-sport capacity [76]. Higher ADI is also associated with increased anxiety, depression, pain interference, fear of reinjury, and reduced readiness to return to sport, all of which are independently linked to poorer rehabilitation adherence and functional outcomes [73, 74, 77–80]. 

Overall, these findings indicate that racial and ethnic disparities in ACL outcomes are driven less by inherent biological differences and more by inequities in access to timely surgery, rehabilitation utilization, and broader socioeconomic conditions that shape outcomes.

## Future Directions

Advancing equity in ACL care will require deliberate shifts in both research design and clinical implementation. Key priorities include improving sex-disaggregated and race- or ethnicity-stratified reporting within prospective studies, while integrating area-level deprivation metrics into outcomes research. Future studies should move beyond descriptive disparities and link demographic and socioeconomic factors to objective outcomes, patient-reported measures, and psychosocial recovery domains. This can help identify modifiable drivers of inequity.

From a clinical perspective, equity-oriented care models should focus on earlier identification of patients at risk for delayed treatment and poor recovery, supported by streamlined referral pathways and reliable access to high-quality rehabilitation services. Rehabilitation and return-to-sport frameworks should be tailored to account for sex-specific physiology, psychological readiness, and structural barriers that influence adherence and recovery.

At the health system level, incorporation of Area and Social Deprivation Index–based screening offers a pragmatic approach to risk stratification and may support targeted care navigation, rehabilitation support, and resource allocation. These efforts shift the focus from documenting disparities to addressing them.

## Conclusion

Recent studies published over the past several years have substantially expanded understanding of how sex, race, and social context influence ACL injury recovery and outcomes. This growing body of work has clarified that, while biological factors contribute to injury risk, differences in surgical timing, rehabilitation access, psychosocial burden, and return-to-sport are central drivers of persistent outcome disparities.

Despite advances, important gaps remain in translating this knowledge into equitable improvements in care. Continued emphasis on integrating biological risk with structural determinants is essential to move the field beyond describing disparities toward developing effective, patient-centered solutions that improve outcomes across diverse populations.

## Key References

- Bruder AM, Culvenor AG, King MG, et al. *Let’s talk about sex (and gender) after ACL injury: a systematic review and meta-analysis of self-reported activity and knee-related outcomes.* Br J Sports Med. 2023.
  - ○ Systematic review/meta-analysis of longitudinal cohort studies showing persistent sex-related differences in activity and KOOS scores after ACL injury and reconstruction, despite comparable objective surgical results.
- Kamatsuki Y, Qvale MS, Steffen K, Wangensteen A, Krosshaug T. *Anatomic Risk Factors for Initial and Secondary Noncontact ACL Injury: A Prospective Cohort Study in 880 Female Elite Athletes.* Am J Sports Med. 2025.
  - ○ Prospective cohort identifying static knee valgus as an independent predictor of primary ACL injury and knee hyperextension for both primary and secondary injury, providing strong evidence on sex-specific biomechanical risk factors.
- Hale ME, Ramos MS, O’Toole RA, et al. *Where Sex Matters for Perioperative and Postoperative ACL Reconstruction Outcomes: A Systematic Review of MOON Literature.* Am J Sports Med. 2026.
  - ○ Systematic review of multi-center cohort data clarifying which ACL reconstruction outcomes differ by biological sex (e.g., strength recovery, return to sport) and which do not (graft tear, contralateral injury), informing interpretations of sex disparities.
- McAleese T, Welch N, King E, et al. *Primary ACL Reconstruction in Level 1 Athletes: Factors Associated With Return to Play*,* Reinjury*,* and Knee Function at 5 Years.* Am J Sports Med. 2025.
  - ○ Longitudinal study of elite athletes examining demographic, graft, and functional predictors of return to play and reinjury at 5 years, providing contemporary benchmarks and highlighting how recovery trajectories differ by sex and activity level.
- Ebert JR, Kneebone L, Edwards P, Radic R, D’Alessandro P. *Return to preinjury pivoting sports after ACL reconstruction differs between males and females.* Knee Surg Sports Traumatol Arthrosc. 2025.
  - ○ Prospective cohort demonstrating lower return-to-sport rates in female athletes and elucidating sex-specific patient-reported barriers, including fear of reinjury and psychosocial factors.
- Haberfield MJ, Donaldson A, Crossley KM, et al. *Considerations for a Women’s Rehabilitation Programme Following ACL Reconstruction: A Concept Mapping Study.* Br J Sports Med. 2026.
  - ○ Mixed-methods study capturing clinician and patient priorities to define rehabilitation domains relevant to women, emphasizing strategies targeting neuromuscular control, psychological readiness, and adaptable care environments.
- Sugimoto D, Myer GD, Foss KDB, Hewett TE. *Specific exercise effects of preventive neuromuscular training intervention on ACL injury risk reduction in young females: meta-analysis and subgroup analysis.* Br J Sports Med. 2015.
  - ○ Meta-analysis demonstrating that neuromuscular training effectively reduces ACL injury risk in female athletes, especially when focused on landing mechanics, strength, and plyometrics.
- Puzzitiello RN, Ludwick LT, Enabulele O, Salzler MJ. *Socioeconomic Disadvantage Is Associated With Delays in ACL Reconstruction and Greater Rates of Concomitant Meniscectomies.* Arthroscopy. 2025.
  - ○ Retrospective cohort showing that higher area-level socioeconomic deprivation independently predicts delayed ACL reconstruction and increased rates of irreparable meniscal injury, linking social context to structural outcome disparities.
- Troyer L, Voshage A, Rucinski K, DeFroda S, Cook JL. *The effects of social determinants of health on anterior cruciate ligament injury recovery.* J Orthop. 2024.
  - ○ Recent analysis linking social determinants, including insurance type and neighborhood factors, with rehabilitation utilization, functional outcomes, and return-to-activity, highlighting mechanisms by which deprivation affects recovery.
- Kiani SN, England P, Gornitzky AL, et al. *Disparate Impact: How Does Social Deprivation Affect Patient-reported Outcomes After ACL Reconstruction in Children and Adolescents?* J Pediatr Orthop. 2023.
  - ○ Pediatric cohort showing that social deprivation correlates with worse PROMIS physical function and pain interference scores after ACL reconstruction, independent of surgical metrics, underscoring the importance of socioeconomic context.

## Data Availability

No datasets were generated or analysed during the current study.
